# The AP-2 Family of Transcription Factors—Still Undervalued Regulators in Gastroenterological Disorders

**DOI:** 10.3390/ijms25179138

**Published:** 2024-08-23

**Authors:** Yi-Jin Yu, Damian Kołat, Żaneta Kałuzińska-Kołat, Zhu Liang, Bo-Qiang Peng, Yun-Feng Zhu, Kai Liu, Jia-Xin Mei, Gang Yu, Wei-Han Zhang, Xiao-Long Chen, Kun Yang, Jian-Kun Hu, Lin-Yong Zhao

**Affiliations:** 1 Department of General Surgery & Laboratory of Gastric Cancer, State Key Laboratory of Biotherapy–Collaborative Innovation Center of Biotherapy and Cancer Center, West China Hospital, Sichuan University, Chengdu 610041, China; yuyijin0314@163.com (Y.-J.Y.);; 2 Gastric Cancer Center, West China Hospital, Sichuan University, Chengdu 610041, China; 3 Department of Functional Genomics, Medical University of Lodz, 90-752 Lodz, Lodzkie, Poland; damian.kolat@stud.umed.lodz.pl (D.K.);; 4 Department of Biomedicine and Experimental Surgery, Medical University of Lodz, 90-136 Lodz, Lodzkie, Poland; 5 Nuffield Department of Medicine, Target Discovery Institute, University of Oxford, Center for Medicines Discovery, Oxford OX1 2JD, UK; 6 Nuffield Department of Medicine, Chinese Academy of Medical Sciences (CAMS), CAMS Oxford Institute (COI), University of Oxford, Oxford OX1 2JD, UK

**Keywords:** AP-2, TFAP2, transcription factors, gastroenterological disorders, gastric cancer, colorectal cancer

## Abstract

Activating enhancer-binding protein 2 (AP-2) is a family of transcription factors (TFs) that play crucial roles in regulating embryonic and oncogenic development. In addition to splice isoforms, five major family members encoded by the *TFAP2A/B/C/D/E* genes have been identified in humans, i.e., AP-2α/β/γ/δ/ε. In general, the first three TFs have been studied more thoroughly than AP-2δ or AP-2ε. Currently, there is a relatively limited body of literature focusing on the AP-2 family in the context of gastroenterological research, and a comprehensive overview of the existing knowledge and recommendations for further research directions is lacking. Herein, we have collected available gastroenterological data on AP-2 TFs, discussed the latest medical applications of each family member, and proposed potential future directions. Research on AP-2 in gastrointestinal tumors has predominantly been focused on the two best-described family members, AP-2α and AP-2γ. Surprisingly, research in the past decade has highlighted the importance of AP-2ε in the drug resistance of gastric cancer (GC) and colorectal cancer (CRC). While numerous questions about gastroenterological disorders await elucidation, the available data undoubtedly open avenues for anti-cancer targeted therapy and overcoming chemotherapy resistance. In addition to gastrointestinal cancers, AP-2 family members (primarily AP-2β and marginally AP-2γ) have been associated with other health issues such as obesity, type 2 diabetes, liver dysfunction, and pseudo-obstruction. On the other hand, AP-2δ has been poorly investigated in gastroenterological disorders, necessitating further research to delineate its role. In conclusion, despite the limited attention given to AP-2 in gastroenterology research, pivotal functions of these transcription factors have started to emerge and warrant further exploration in the future.

## 1. Introduction

**Figure 1:**
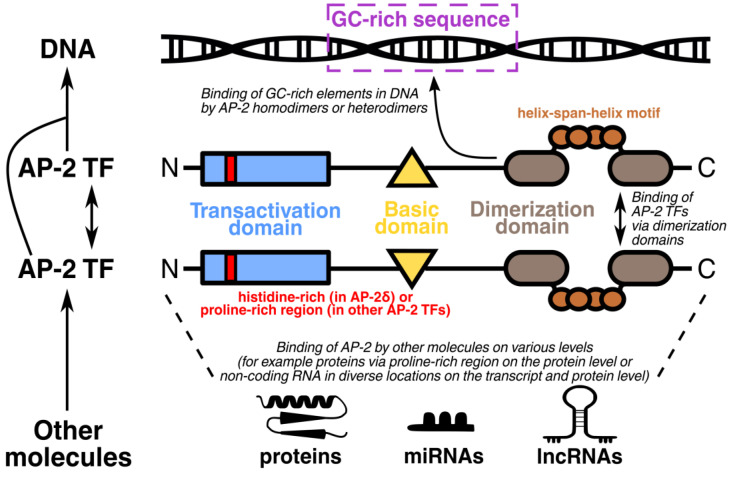
Structure and interactions of AP-2 transcription factors. Each AP-2 family representative contains transactivation, basic, and dimerization domains but AP-2δ is characterized by unique amino-terminal sequences overlapping with the transactivation domain. AP-2 homodimers and heterodimers bind to guanine and cytosine-rich (GC-rich) DNA elements, such as GCCN_3/4_GGC, GCCN_3/4_GGG, or CCCCAGGC. On the other hand, AP-2 may be bound by other molecules such as proteins, micro RNA (miRNA), and long non-coding RNA (lncRNA).

Activating enhancer-binding protein 2 (AP-2) is a family of transcription factors (TFs) important for orchestrating embryonic and oncogenic development. Excluding splice isoforms, five main family members encoded by the *TFAP2A/B/C/D/E* genes were identified in humans, namely AP-2α/β/γ/δ/ε. In contrast to *TFAP2A*, *TFAP2B*, and *TFAP2D* which are located in chromosome 6, *TFAP2C* and *TFAP2E* are encoded on chromosome 20 and 1, respectively. Although the entire AP-2 family is characterized by highly conserved domains, such as the basic domain and dimerization domain containing the helix-span-helix motif, the proline-rich region in the transactivation domain of AP-2α/β/γ/ε is replaced by a unique histidine-rich region in AP-2δ [1,2]. When interacting with their target genes, AP-2 homodimers and heterodimers typically bind to cis-regulatory elements rich in guanine and cytosine, such as GCCN_3/4_GGC, GCCN_3/4_GGG, or CCCCAGGC (Figure 1) [3]. Based on the UniProt Knowledgebase [4], AP-2α/β/γ/δ/ε canonical proteins are composed of 437, 460, 450, 452, and 442 amino acids, respectively. Three isoforms of AP-2α range from 365 to 433 in length, whereas AP-2β and AP-2γ have only one isoform each (469 and 281 amino acids, respectively). No alternative splice isoforms are currently known for AP-2δ and AP-2ε. Even though AP-2 transcription factors were discovered decades ago [5,6,7,8,9], the entire family has not received extensive research in the field of gastroenterological disorders relative to other systemic diseases, necessitating a more systematic approach and guidance for future research directions.

Gastroenterological disorders encompass a range of conditions affecting the digestive tract, including infectious diseases (such as acute appendicitis and infectious diarrhea), non-infectious diseases (such as inflammatory bowel disease, peptic ulcers, and gastrointestinal perforations), and malignant diseases (such as gastric cancer, colorectal cancer, and esophageal cancer) [10]. These diseases are highly prevalent in the general population, with malignancies (particularly colorectal cancer and gastric cancer) posing substantial challenges to global healthcare systems. Recent epidemiological data indicate that colorectal cancer is the third most common malignancy worldwide and the second leading cause of cancer-related mortality [11], while gastric cancer is the fifth most common malignancy and the third leading cause of cancer-related deaths globally [11].

A few studies have demonstrated that, except for the almost entirely unexplored AP-2δ, the remaining members of the AP-2 family play pivotal roles in various dimensions of gastroenterological disorders, encompassing critical aspects such as growth regulation, metabolic modulation, carcinogenesis facilitation, and chemoresistance, particularly in the context of gastrointestinal malignancies. As our understanding of intricate functional mechanisms of AP-2 family members deepens, and in conjunction with the integration of cutting-edge methodologies, it is reasonable to expect that forthcoming investigations will increasingly be focused on this specific issue, thus propelling and catalyzing advancements within the field of gastroenterology. Hence, we have gathered available data on AP-2 in gastroenterological disorders alongside its latest medical applications, hoping to promote research on this issue and serve as a reference for future research directions. 

A literature search was performed in February-March 2024 via PubMed with the following terms: (1) TFAP2 and gastric, (2) TFAP2 and colorectal, (3) TFAP2 and intestine, (4) TFAP2 and stomach, (5) AP-2 and gastric, (6) AP-2 and colorectal, (7) AP-2 and intestine, (8) AP-2 and stomach. In the above query conditions, we replaced “TFAP2” and ”AP-2” with “TFAP2A”, “TFAP2B”, “TFAP2C”, “TFAP2D”, “TFAP2E”, “TFAP2α”, “TFAP2β”, “TFAP2γ”, “TFAP2δ”, “TFAP2ε” and “AP-2A”, “AP-2B”, “AP-2C”, “AP-2D”, “AP-2E”, “AP-2α”, “AP-2β”, “AP-2γ”, “AP-2δ”, “AP-2ε”, respectively, for query.

## 2. The AP-2 Family in Gastric Cancer (GC)

In recent years, a surge in research has accentuated the importance of transcription factors in the cascade of oncogenic events. The roles of AP-2α, AP-2γ, and AP-2ε have emerged as focal points in understanding the molecular underpinnings of GC, painting a multifaceted picture of their roles in tumorigenesis, cancer progression, and potential therapeutic interventions.

Exploring the involvement of AP-2α in GC revealed a spectrum of oncogenic activities. This TF promotes cell proliferation, migration, and invasion, establishing its oncogenic role in both GC and other carcinomas [12]. For example, epigallocatechin gallate (EGCG) can effectively inhibit the pathway involving AP-2α and vascular endothelial growth factor (VEGF), leading to the downregulation of downstream molecules such as MDR-1 and P-gp, ultimately resulting in the inhibition of proliferation and reversal of 5-fluorouracil (5-FU) resistance in GC cells [13]. Furthermore, the tumor suppressor ZNF471 and the co-repressor factor KAP1 work in synergy to suppress the *TFAP2A* promoter by inducing an increase in histone H3 trimethylation on lysine 9 (H3K9me3), thereby inhibiting proliferation, migration, and invasion within GCs [14]. Moreover, AP-2α facilitates the epithelial–mesenchymal transition (EMT) in gastric cancer with amplification of the erb-b2 receptor tyrosine kinase 2 (ERBB2), whose molecular characteristics highlight AP-2α as a potential target for individuals resistant to anti-ERBB2 therapies, thereby expanding the horizon of AP-2α-targeted treatments [15]. Nevertheless, the AP-2α narrative is not without contradictions. While most studies indicate that this TF primarily plays an oncogenic role in GC, others have proposed a correlation between low AP-2α expression and advanced tumor stage or a poorer prognosis [16,17]. Some data for *TFAP2A-AS1*, the gene encoding a long non-coding RNA located on a DNA strand other than *TFAP2A*, are also available. It has been confirmed that *TFAP2A-AS1* exerts an inhibitory effect on the proliferation and migration of GC cells, and this action is positively orchestrated by its upstream regulatory protein KLF15 through its modulation of the downstream target, i.e., miR-3657 [18]. This dichotomy highlights the necessity for more comprehensive research to elucidate the subtle distinctions in the role of AP-2α within the realm of GC. 

AP-2γ can also play a carcinogenic role in GC. Based on both in vitro and in vivo data, this TF is recruited by a long non-coding RNA LINC00857, which enhances the expression of the FAT1 protein to promote tumorigenesis and EMT [19]. Another study indicated that *TFAP2C* methylation is observed in primary GC, and 30-fold up-regulation of this gene was noted following treatment with the demethylating agent 5-aza-2′-deoxycytidine [20]. Despite the role of AP-2γ in the carcinogenic mechanisms of GC, limited research has been conducted on this topic, necessitating further investigation.

Research on AP-2δ relative to other family members is regrettably limited in gastroenterology, similar to the general view. The only gastroenterological clue was found in our recent study, in which stomach tumors were among the malignancies most susceptible to AP-2δ mutations, with these alterations correlating to the expression of cancer hallmark genes and known drug targets [2].

**Figure 2:**
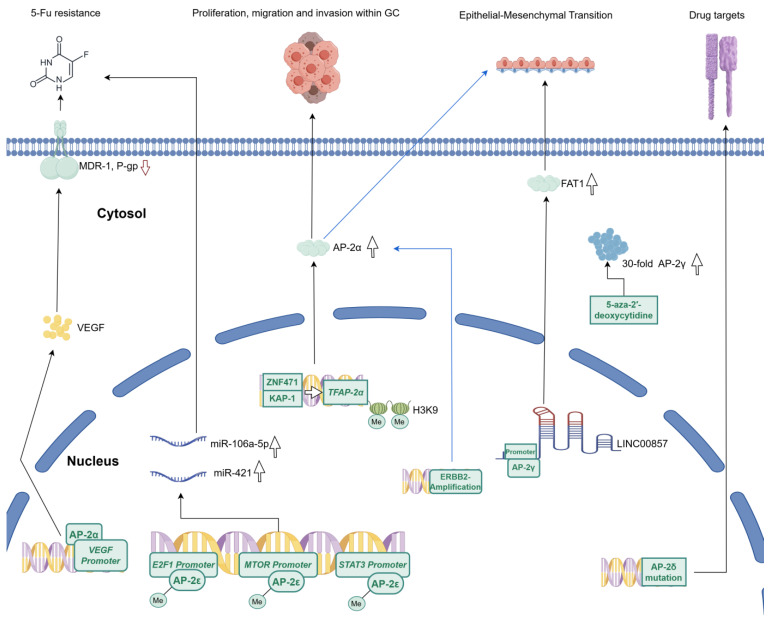
The role of the AP-2 family in GC. The AP-2 family plays a regulatory role in GC through the VEGF pathway, up-regulation of miRNA, specific expression, gene mutation, etc. Notably, only the functions that have an explicit molecular mechanism are listed here.

Currently, the primary direction of research related to AP-2ε concerns its involvement in GC chemoresistance [21]. *TFAP2E* hypermethylation is closely associated with increased chemoresistance to 5-FU in GC [22]; this status may serve as a potential predictive factor for treatment response. Given the usefulness of AP-2ε in chemotherapy, the DNA methyltransferase inhibitors (such as decitabine encapsulated in nanoparticles) have been employed to ameliorate chemoresistance in GC via enhanced demethylating activity towards *TFAP2E* and subsequent re-expression [23,24]. However, the mechanisms of chemoresistance involving AP-2ε in GC appear intricate and differ from those in colorectal cancer [25]. Some GC-related data suggest that this difference may be associated with the release of microRNA-containing exosomes in response to *TFAP2E* hypermethylation. It was hypothesized that two microRNAs, miR-106a-5p and miR-421, are dependent on *TFAP2E* methylation status and orchestrate the expression of genes such as *E2F1*, *STAT3*, and *MTOR*, which are associated with chemoresistance to 5-FU in GC [26]. Unfortunately, in addition to the aforementioned arguments, research on the resistance mechanisms of AP-2ε is rare and a unified conclusion has not been reached, necessitating further exploration. A graphical schematic of the molecular mechanism of the AP-2 family in GC is summarized in Figure 2.

## 3. The AP-2 Family in Colorectal Cancer (CRC)

CRC continues to pose a global health challenge due to the complex molecular mechanisms governing its initiation and progression. Within the extensive array of molecular contributors, the AP-2 family has emerged as a pivotal regulator in CRC, with AP-2α, AP-2γ, and AP-2ε manifesting critical and well-described regulatory roles.

Interestingly, unlike its tumorigenic role in GC, AP-2α predominantly acts against CRC, which manifests as a decrease in tumor growth, EMT inhibition, and cell cycle arrest. In CRC, AP-2α interacts with the adenomatous polyposis coli (APC) protein, converting nuclear β-catenin into an inactive form, further reducing its association with the TCF/LEF transcription factors, ultimately disrupting the Wnt/β-catenin pathway and inhibiting EMT progression [27]. Moreover, AP-2α loss results in the dysregulation of E-cadherin and matrix metalloproteinase-9, increasing the tumorigenicity of colorectal cancer cells in vivo [28]. Another study indicated that AP-2α is essential for the PTEN-mediated inhibition of proliferation and the cell cycle in response to the ganglioside GM3 [29]. Additionally, AP-2α mediates the anti-tumor effects of small ubiquitin-like modifier (SUMO) inhibitors by downregulating CD44 and MMP14, restraining tumor stemness [30]. At the transcriptional level, AP-2α and AP-2γ can bind to the promoter of *CDKN1A* (also known as p21), enhancing its expression and leading to reduced cyclin D1, dephosphorylation of the retinoblastoma protein, inactivation of the E2F transcription factor, and ultimately cell cycle arrest [31]. Furthermore, AP-2α induces cell cycle arrest and apoptosis in colorectal cancer cells in both a p53-dependent and p53-independent manner [32,33]. In vivo research on APC^min/+^ mice revealed that AP-2α delivery suppresses intestinal polyp formation and exerts tumor-suppressive effects on the gastrointestinal tract [34]. The authors of the same study suggested AP-2α as a valuable tool for anti-cancer interventions in patients with dysregulated Wnt signaling. However, AP-2α is not entirely beneficial in CRC. For example, lactate released by CRC cells can induce the nuclear translocation of AP-2α in tumor-associated macrophages (TAMs), leading to the transcriptional upregulation of ELK1 by AP-2α. This promotes the expression of signal regulatory protein α (SIRPα), which can suppress the phagocytic activity of TAMs to establish an immune evasive microenvironment [35]. Although the role of AP-2α in colorectal cancer remains a subject of debate, its value and dynamism need to be explored in further research, as AP-2α could serve as a target for CRC treatment.

AP-2γ continues to play an oncogenic role in CRC by promoting proliferation, migration, and invasion via activation of the PI3K/AKT signaling pathway. This mechanism involves a complex interplay where AP-2γ enhances the expression of the circular RNA circIL4R, which, in turn, interacts with miR-761 to increase the expression of tripartite motif-containing 29 (TRIM29). Afterwards, the TRIM29 protein induces proteasome-mediated degradation of the leucine-rich repeat protein phosphatase 1 (PHLPP1), consequently activating the PI3K/AKT signaling pathway [36]. Moreover, AP-2γ regulates the stemness of colorectal cancer cells through various pathways, enhancing the expression of stem cell-related factors such as Nanog, BMI-1, OCT4, and SOX2, as well as the phenotypic traits of cancer stem cells, thereby promoting tumorigenesis [37]. Regarding chemoresistance, AP-2γ promotes the expression of YAP1 and TAZ, inhibiting the Hippo signaling pathway and mediating the resistance of CRC to 5-FU chemotherapy [37]. This finding reveals potential avenues to overcome chemotherapy resistance, which remains a challenge in colorectal cancer treatment.

The availability of gastroenterology data for AP-2δ is limited to our recent study [2]. As mentioned in the previous section, some cancer types were more susceptible to AP-2δ mutations, which correlated to the expression of cancer hallmark genes and known drug targets. Together with the aforementioned stomach tumors, colon malignancies were among these cancer types. Although AP-2δ remains uncharted territory even outside the topic discussed, its relatively high expression in, e.g., the small intestine, suggests that this TF has essential gastroenterological functions that are yet to be discovered. This hypothesis can be supported by the distinct expression pattern of AP-2δ relative to AP-2β, which are both located in the exact cytological location (chromosome 6p12.3), but the latter is mainly expressed in adult kidney tissue where AP-2δ is undetectable [8].

Similar to the role of AP-2ε in GC, *TFAP2E* hypermethylation also mediates chemotherapy resistance in CRC and can serve as a predictive factor for the 5-FU response [38]. Notably, *TFAP2E* methylation is an independent predictor of early recurrence, especially in stage II CRC patients [39]. Although AP-2ε tends to act as a tumor suppressor [1], some CRC-related data link *TFAP2E* hypermethylation with low invasion, reduced lymph node metastasis, and favorable prognosis [40]. These inconsistencies may depend on whether the CRC has a *BRAF* mutation or is wild-type in this context [41]. AP-2ε–mediated chemoresistance mechanisms in CRC have been explored. *TFAP2E* hypermethylation reduces AP-2ε expression, weakening its inhibitory effect on cyclin-dependent kinase 4 and thereby inducing resistance to 5-FU [42,43]. Another study suggested that AP-2ε intensifies Wnt/β-catenin signaling via transcriptional inhibition of the Dickkopf-related protein 4 (DKK4), which antagonistically occupies the Wnt receptor and is known to promote 5-FU chemoresistance [44]. Future reports should continue exploring the relevance of AP-2ε in overcoming drug resistance.

**Figure 3:**
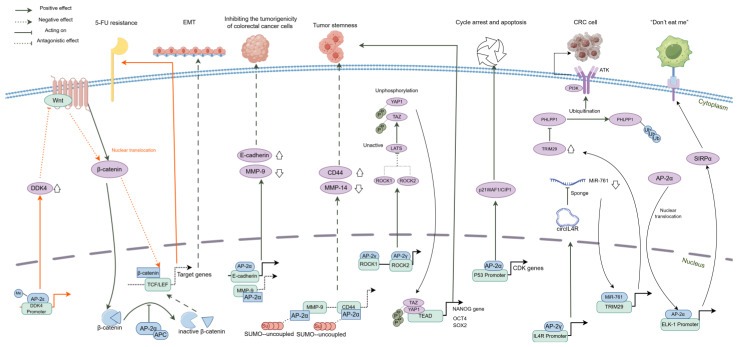
The regulatory modes of AP-2 family on downstream targets in CRC.

In addition to the recognized oncogenic function of AP-2γ, current research into AP-2α/ε in colorectal cancer has produced conflicting findings, leaving their exact mechanistic roles and impacts subject to further validation. Additionally, the dichotomous nature of these transcription factors must be carefully weighed in the context of their clinical therapeutic applications to derive more accurate and nuanced conclusions. A graphical schematic of the molecular mechanism of the AP-2 family in CRC is summarized in Figure 3.

## 4. The AP-2 Family in Other Gastroenterological Disorders

A few investigations have investigated the involvement of AP-2 family members in metabolic and gastroenterological disorders, revealing their importance in the pathological landscape. We delineated the emerging evidence highlighting the roles of AP-2 family members in obesity, type 2 diabetes, gastrointestinal dysmotility, and esophageal squamous cell cancer, thereby suggesting a potential therapeutic avenue for these disorders.

AP-2β has been shown to be associated with obesity and type 2 diabetes [45]. Its genetic variants are significantly related to body mass index and weight loss in high-fat diet groups, emphasizing its potential role in metabolic health [46,47]. Recent findings have implicated AP-2β in pediatric intestinal pseudo-obstruction, a severe gastrointestinal dysmotility disorder. Haploinsufficiency of *TFAP2B* reduces the number of enteric neurons and delays gastrointestinal transit time, suggesting that AP-2β is a novel candidate for understanding and potentially treating gastrointestinal dysmotility disorders [48]. 

The expression of AP-2γ in the intestine has been associated with cellular hyperplasia and suppression of terminal differentiation, hinting at its important role in intestinal homeostasis. Its expression leads to the activation of the SOX9 transcription factor and induces the proliferation of active epithelial progenitor cells, resulting in the defective development of mucosal crypt cells and the loss of mucosal differentiation [49]. Transgenic overexpression research has provided a model for studying liver failure and intestinal dysplasia, emphasizing the importance of AP-2γ in gastroenterological health. 

Finally, AP-2ε was identified as a definite risk marker for esophageal squamous cell cancer based on the significant differences in *TFAP2E* methylation levels between the groups with high and intermediate risk of cancer [25].

To recapitulate all of the above sections, the role of AP-2 transcription factors in GC, CRC, and other gastroenterological disorders is summarized in Table 1. The AP-2 family is pivotal in the pathophysiology of myriad metabolic and gastroenterological disorders, providing enhanced insights into these diseases and leading to new avenues for therapeutic intervention. Despite the current relative paucity of related research, the emerging clinical therapeutic potential of these molecules is becoming increasingly apparent. Consequently, there is a compelling need for more comprehensive research endeavors aimed at unraveling the intricate mechanisms by which members of the AP-2 family exert their multifaceted effects, a pursuit essential for advancing targeted and effective clinical treatments in these domains.

## 5. Conclusions

We have summarized the role of the AP-2 family in gastroenterological disorders, and most of the related data concern GC and CRC, but some related reports extend beyond the subject of cancer. Although research on the AP-2 family in gastroenterology is still relatively scarce, existing studies have presented various phenotypic aspects and analyzed presumptive mechanisms, revealing strong potential for the application of these TFs in gastroenterological disorders.

The oncogenic roles of AP-2α and AP-2γ have been extensively investigated in GC and CRC, as they regulate tumor progression through multiple pathways, forming complex regulatory networks. The widely recognized chemotherapy resistance caused by hypermethylation of the AP-2ε-encoding gene holds promise for enhancing cancer treatment or overcoming resistance. Research on AP-2δ is limited, necessitating further exploration to elucidate its role in gastroenterological disorders. AP-2β is involved in obesity, type 2 diabetes, and gastrointestinal dysfunction, which provides a broader scope for research to reveal new therapeutic targets.

Although the AP-2 family has gradually shown therapeutic potential, different subtypes of AP-2 (such as AP-2α and AP-2γ) can have opposite effects within the same disease and varying roles in different diseases. In terms of translational application, comprehensive clinical data and considerations are still required. Moreover, related research tends to focus more on tumor cells, with studies on the extracellular matrix and immune microenvironment still lacking, necessitating further investigation. Additionally, research on other gastroenterological disorders is limited to a few conditions, with currently no studies regarding inflammatory bowel disease, intestinal polyps, and diverticulosis. Based on existing studies, AP-2 remains undervalued, with the therapeutic usefulness of AP-2ε not being properly translated to clinical settings, which necessitates extensive further research.

Conclusively, in targeted therapy and chemotherapy for several cancers, AP-2 can serve as a predictive or regulatory factor, yet its clinical importance is often overlooked. For bridging the gap between the molecular functions of this TF family and practical applications, additional research is needed to reveal the factors initiating alterations in AP-2 expression, outline the regulatory networks involved in the interactions of AP-2 TFs with other molecules, and establish the clinical relevance of AP-2. Such efforts will contribute to harnessing the full potential of AP-2 in clinical and translational settings.

## Figures and Tables

**Table 1:** The role of AP-2 transcription factors in gastroenterological disorders.

AP-2 Member	Biological Outcome and Related Mechanism (If Known)	Role of AP-2	Ref.
Gastric cancer			
AP-2α	Epigallocatechin gallate downregulates the AP-2α/VEGF pathway, inhibiting proliferation and decreasing 5-FU resistance.	Unfavorable	[13]
	ZNF471 and KAP1 induce TFAP2A promoter hypermethylation, which reduces proliferation, migration, and invasion.	Unfavorable	[14]
	AP-2α facilitates epithelial–mesenchymal transition in ERBB2Amp-type gastric cancer.	Unfavorable	[15]
	Low AP-2α expression correlates with advanced tumor stage and a poorer prognosis.	Beneficial	[16,17]
AP-2γ	AP-2γ is recruited by LINC00857, which enhances FAT1 and promotes tumorigenesis, as well as epithelial–mesenchymal transition.	Unfavorable	[19]
	30-fold upregulation of TFAP2C was noted after treatment with 5-aza-2′-deoxycytidine.	Inconclusive	[20]
AP-2δ	AP-2δ mutations correlate with the expression of cancer hallmark genes and drug targets.	Inconclusive	[2]
AP-2ε	TFAP2E hypermethylation is associated with increased chemoresistance to 5-FU; miR-106a-5p and miR-421 are dependent on TFAP2E methylation status and regulate the expression of E2F1, STAT3, and MTOR, which are associated with 5-FU chemoresistance.	Beneficial	[22,26]
Colorectal cancer			
AP-2α	AP-2α interacts with APC, disrupting the Wnt/β-catenin pathway and inhibiting EMT.	Beneficial	[27]
	AP-2α loss dysregulates E-cadherin and MMP-9, increasing the tumorigenicity.	Beneficial	[28]
	AP-2α is essential for the PTEN-mediated inhibition of proliferation and cell cycle.	Beneficial	[29]
	AP-2α mediates anti-tumor effects of SUMO inhibitors by downregulating CD44 and MMP14, restraining tumor stemness.	Beneficial	[30]
	AP-2α induces cell cycle arrest and apoptosis (p53-dependent or p53-independent manner).	Beneficial	[32,33]
	AP-2α suppresses intestinal polyp formation and exerts tumor-suppressive effects.	Beneficial	[34]
	Lactate released by tumor cells can induce the nuclear translocation of AP-2α in TAMs, leading to upregulation of ELK1 and SIRPα, thereby decreasing the phagocytic activity of TAMs and ensuring immune evasion.	Unfavorable	[35]
AP-2γ	AP-2γ enhances CircIL4R, whichactivates the PI3K/AKT signaling pathway via the miR-761/TRIM29/PHLPP1 axis, intensifying proliferation, migration, and invasion.	Unfavorable	[36]
	AP-2γ enhances the expression of Nanog, BMI-1, OCT4, and SOX2, regulating phenotypic traits of stem cells and promoting tumorigenesis.	Unfavorable	[37]
	AP-2γ expression is positively associated with YAP1 and TAZ, which inhibits the Hippo pathway and increases 5-FU resistance.	Unfavorable	[37]
AP-2δ	AP-2δ mutations correlate with the expression of cancer hallmark genes and drug targets.	Inconclusive	[2]
AP-2ε	TFAP2E hypermethylation is related to low invasion, reduced lymph node metastasis, and favorable prognosis.	Unfavorable	[40]
	AP-2ε inhibits cyclin-dependent kinase 4 and decreases resistance to 5-FU.	Beneficial	[42,43]
	AP-2ε intensifies Wnt/β-catenin signaling via inhibition of DKK4, which is known to promote 5-FU chemoresistance.	Beneficial	[44]
Other gastroenterological disorders			
AP-2β	Genetic variants of AP-2β are related to BMI and weight loss in high-fat diet groups.	Inconclusive	[46,47]
	TFAP2B haploinsufficiency decimates enteric neurons and delays gastrointestinal transit time, causing intestinal pseudo-obstruction.	Beneficial	[48]
AP-2γ	AP-2γ induces SOX9 and intensifies epithelial progenitor cell expansion, resulting in liver failure and intestinal dysplasia.	Unfavorable	[49]
AP-2ε	TFAP2E methylation discriminates groups with various risks of cancer; a putative risk marker for esophageal squamous cell cancer.	Inconclusive	[25]

Ref.—reference; 5-FU—5-fluorouracil; EMT—epithelial–mesenchymal transition; SUMO—small ubiquitin-like modifier; TAMs—tumor-associated macrophages; BMI—body mass index.
